# Does the shape of the electric pulse matter in electroporation?

**DOI:** 10.3389/fonc.2022.958128

**Published:** 2022-09-14

**Authors:** Vitalij Novickij, Nina Rembiałkowska, Wojciech Szlasa, Julita Kulbacka

**Affiliations:** ^1^ Faculty of Electronics, Vilnius Gediminas Technical University (Vilnius TECH), Vilnius, Lithuania; ^2^ Department of Molecular and Cellular Biology, Faculty of Pharmacy, Wroclaw Medical University, Wroclaw, Poland; ^3^ Faculty of Medicine, Wroclaw Medical University, Wroclaw, Poland

**Keywords:** electric pulse, pulse shape, electroporation, permeabilization, pulse frequency

## Abstract

Electric pulses are widely used in biology, medicine, industry, and food processing. Numerous studies indicate that electroporation (EP) is a pulse-dependent process, and the electric pulse shape and duration strongly determine permeabilization efficacy. EP protocols are precisely planned in terms of the size and charge of the molecules, which will be delivered to the cell. In reversible and irreversible EP applications, rectangular or sine, polar or bipolar pulses are commonly used. The usage of pulses of the asymmetric shape is still limited to high voltage and low voltage (HV/LV) sequences in the context of gene delivery, while EP-based applications of ultra-short asymmetric pulses are just starting to emerge. This review emphasizes the importance and role of the pulse shape for membrane permeabilization by EP.

## The first waveforms for electroporation

Electroporation is a pulse-dependent phenomenon; therefore, the treatment can be controlled by changing the waveform, duration, a number of pulses, or burst delivery frequencies. Since the progress of using and developing new pulsing protocols depends on the electronics and the semiconductor technology behind the waveform generators, the respective evolution of electroporation in the past 50 years is also electronics-dependent.

**Figure 1:**
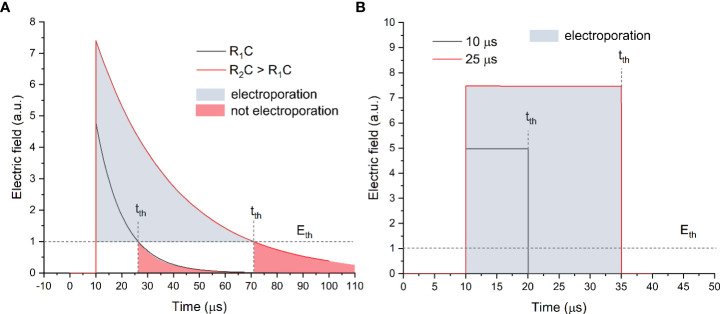
The exponential decay **(A)** and square wave **(B)** pulse waveforms, where t_th_ is the time when the amplitude drop below the electric field value required for electroporation (E_th_).

Back in the day, the pulse generators (electroporators) frequently employed spark gaps as power switches to form the desired signal (1–4). As a result, in the case of capacitor discharge systems, the waveform featured an exponential decay form (1, 5) (see **Figure 1A**).

As can be seen in **Figure 1A**, the exponential decay waveform has a decaying tail, which depends on the RC parameters of the load. In order to control the duration of the pulse, a resistance (R) should be connected in series and/or the impedance of the biological load should be altered (which is not usually the case). Such an approach was used to generate pulses ranging from several microseconds to hundreds of milliseconds and, thus, trigger various rates of electroporation. Starting from the early 1980s, the exponential decay waveform electroporators feature solid-state electronics and employ silicon-controlled rectifiers to substitute the spark gap switches (6). However, the popularity of such a waveform in electroporation is constantly declining due to the complex control of the pulse duration and the energy losses. Electroporation is a threshold-type phenomenon; therefore, a certain electric field (Eth) is required for each type of cell to trigger plasma membrane permeabilization (7, 8). In the case of exponential decay pulses (refer to **Figure 1A**), the electric field amplitude drops below the E_th_ at a time moment t_th_, which implies that the energy is being used on other processes such as electrophoresis, electrolysis, and Joule heating rather than electroporation. Therefore, starting from the early 1990s, the tendency to employ square waves to trigger electroporation was observed (9–11). Indeed, due to sharp rise and fall times (refer to **Figure 1B**), the pulse resembles a square wave; therefore, the losses during the transient changes of the electric field are minimal; i.e., the electric field is above the E_th_ during the whole pulse. In the early days, the square wave pulsing was limited to transmission line systems (2, 12) until the mass availability of high power and high voltage transistors and the occurrence of commercial square wave electroporators.

Later, the square wave pulses became the dominant waveform to be used in electroporation studies. With the development of fast MOSFET and IGBT switches, the parametric protocol design started to feature unipolar and bipolar pulse delivery, duration control from nanoseconds to hundreds of milliseconds, and flexible frequency control. Some studies employ sine wave (13, 14) or bell-shape pulses (15) (16) to grasp the new opportunities in electroporation, but none of them have gone mainstream yet. So far, it has been established that square wave pulses are more effective for electroporation than the sinusoidal ones or other waveforms with slower rise and fall times (17).

**Figure 2:**
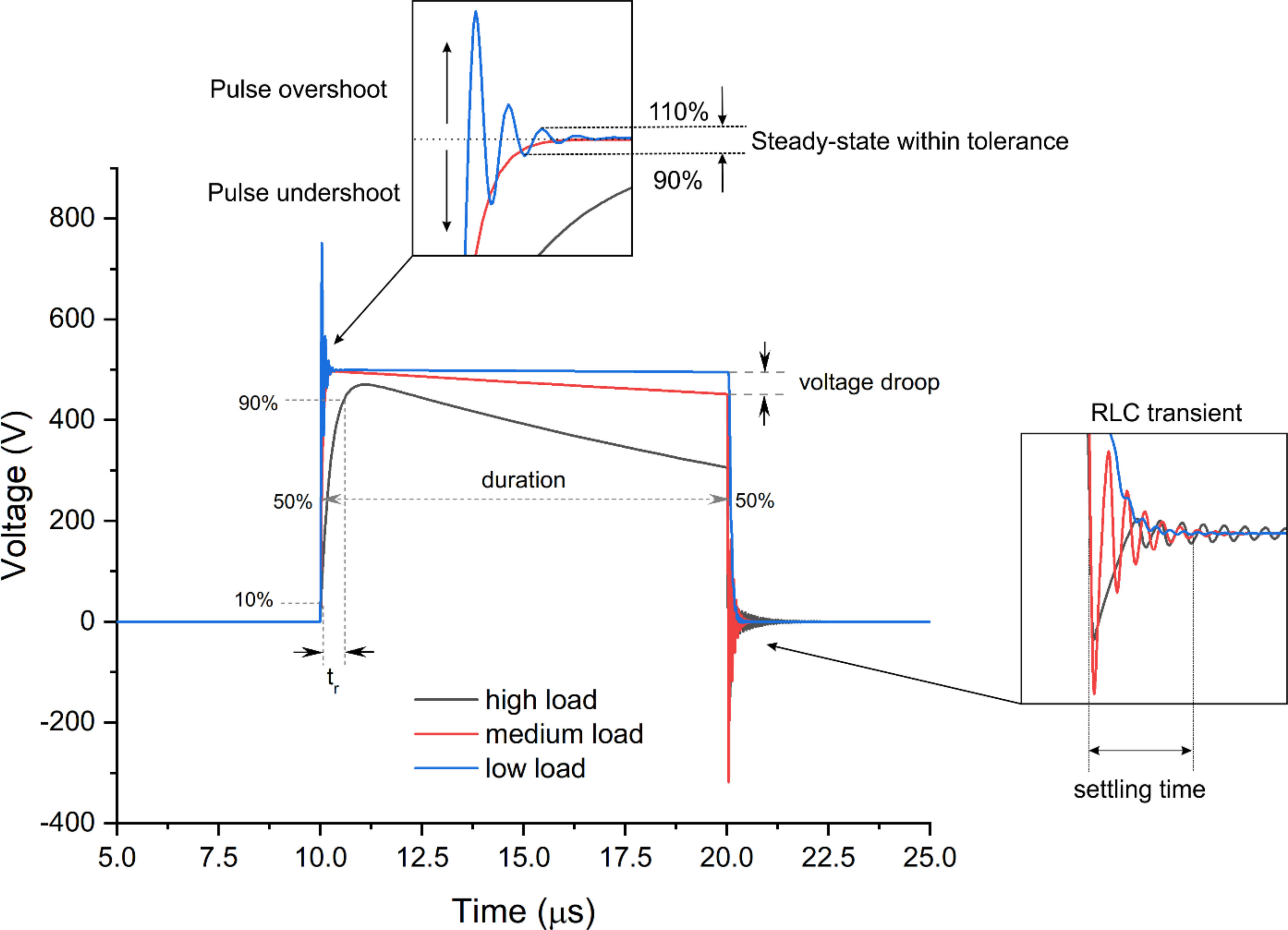
An example of typical transient processes occurrent in electroporation systems during pulse delivery, where t_r_ is the pulse rise time and RLC is the resistor/inductor/capacitor circuit.

Nevertheless, the square wave pulse features a square form only on paper. In reality, various transient processes occur, resulting in significant changes in the ideal shape. An example of transient processes for a 10-μs pulse is shown in **Figure 2**.

It can be seen that the shape of the pulse is load-dependent, which is dramatic in the context of electroporation. During electroporation, the bioimpedance is dynamic (18, 19), and the load of the generator both *in vitro* and *in vivo* depends on the experimental conditions (i.e., electrode gap, contact area, buffer or tissue conductivity, etc.). It implies that the same generator with the same pulse setting will deliver different pulses depending on the experiment or clinical setting. Considering the capacitive nature of the cell membrane, which is charged during the pulse to a specific threshold transmembrane potential (20), such differences in pulse shape may have a significant effect on the electroporation process and, in the end, on the treatment outcome. Therefore, adequate metrology for electroporation experiments and clinical application is required.

It should be noted that it is not the biological load that changes the pulse but rather the non-ideality of the inner electronic components of the generator (electroporator), which is employed in the treatment. Basically, the dominant majority of modern square wave electroporators will feature three major components highly influencing the pulse generation (1: the energy storage capacitor; 2: the semiconductor switch, and 3: the distributed parasitic capacitance and inductance of the circuit) (21, 22). The energy stored in the capacitor will influence how well the generator will handle various loads and pulse durations without a significant droop of voltage (refer to **Figure 2**). The bigger the capacitor, the smaller the droop; however, it is always a trade-off between the size of the electroporator, its price, and pulsing flexibility. Nowadays, a droop of voltage up to 10%–20% is usually tolerated in electroporation studies (23), while it should be understood that 10% is still a significant change in the electric field for a threshold-type phenomenon like electroporation.

**Figure 3:**
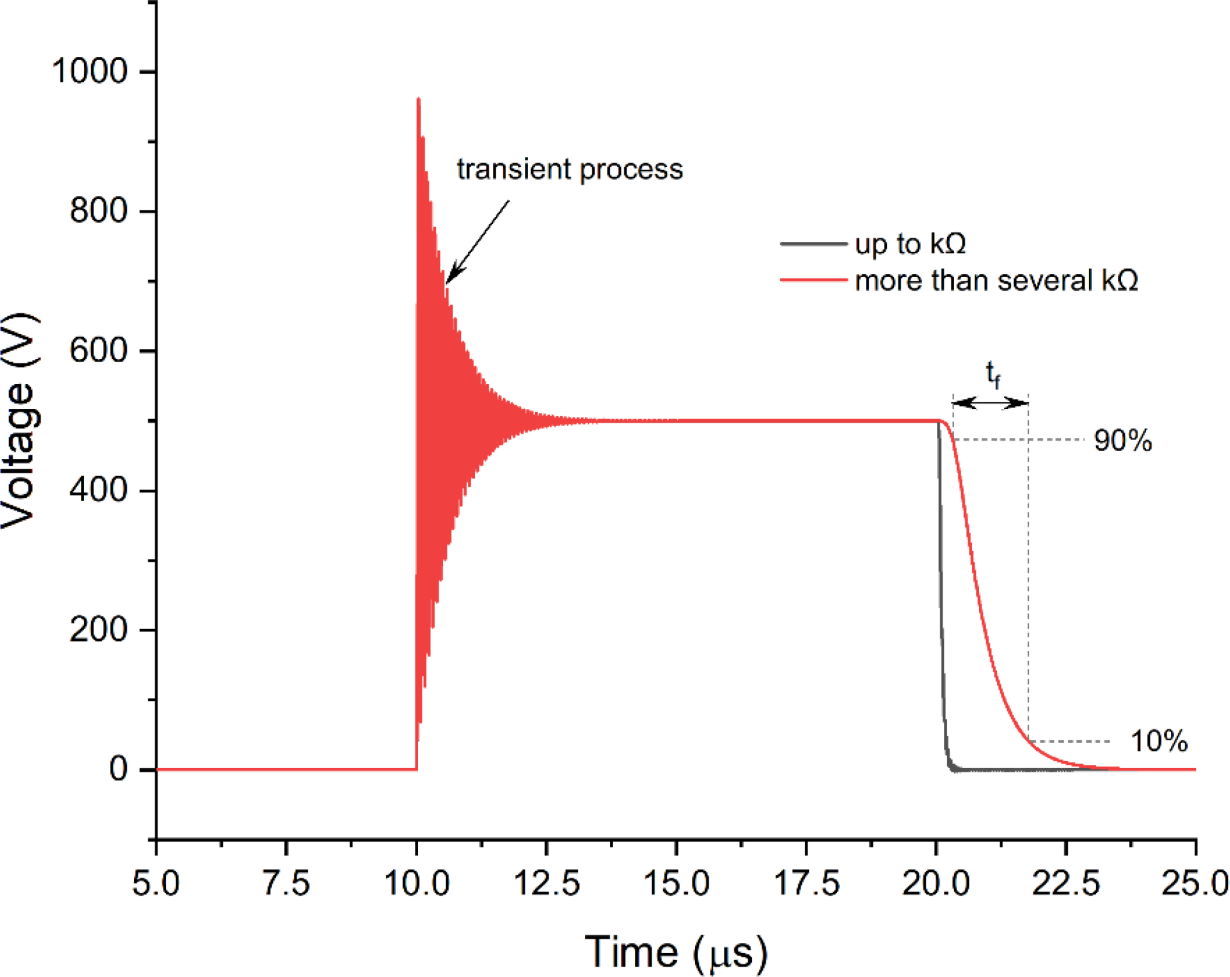
An example of the typical influence of the load impedance on pulse shape.

The semiconductors also play a role due to the dependence of the dynamic characteristics of the switches on the load. However, rise times (t_r_, **Figure 2**) and fall times (t_f_, **Figure 3**) are no longer an issue since modern transistors offer switching times in the sub-50-ns range, which is significantly faster than the polarization time of typical cells (24). An exception is the extremely low loads in the kOhm range, which may alter the rise time transient and fall times of the MOSFET or IGBT transistors (**Figure 3**).

A typical solution is to shunt the load with low parallel resistance (25) or introduce active circuitry to form the pulse independently on the load (26). In the first case, such a solution will influence higher voltage droop, and in the second case, the complexity of the circuit and its driving increase dramatically. However, it is obvious that if the high bioimpedance is left unmatched or uncompensated, the differences in pulse shape inevitably will alter the cellular response to the treatment.

The distributed parasitic RLC parameters are inevitable in any electrical circuit. The capacitive component is highly responsible for the rise-time transient (during low loads), while the parasitic inductance starts to alter the pulse significantly with high loads. In **Figure 2**, it can be seen that with the high load, the rise time becomes longer, and an RLC transient is present at the end of the pulse. Each generator has unique distributed parasitic parameters; therefore, the prediction of the exact pulse shape is complex and usually evaluated empirically. Lastly, it should be noted that the shorter the pulse, the harder it is to ensure a square waveform. It is typical for pulses in the sub-50-ns range that the waveform resembles a bell shape rather than a square wave (15), which is a limitation of state-of-the-art high-power semiconductors. Faster rise and fall times (sub-nanosecond range) can be ensured for low-power pulses, which are applicable in microscale electroporation studies (i.e., using lab-on-chip or microscope coverslips with micrometer-gap electrodes) (27).

With the understanding of the limitations of square wave pulse forming, the effects of various square wave pulses are further reviewed.

## Unipolar square wave pulses

With the understanding of the benefits of square wave pulsing and considering the limitations of available semiconductors three decades ago, the micro-millisecond duration pulses started to dominate the field. In the context of cancer treatment, two main treatment approaches, including tissue ablation or irreversible electroporation (IRE) and electrochemotherapy, were heavily introduced into the clinics (28–31). In both cases, the microsecond and millisecond pulses were employed. Electrochemotherapy was more standardized, often featuring a series of 100-µs pulses to be used in the studies (32–34), later forming a base for ESOPE protocols (European Standard Operating Procedures of Electrochemotherapy) (35). In the case of IRE, the protocols were more flexible, including 100-µs procedures (36, 37). However, it also featured millisecond range pulses (29) and a variety of other microsecond and millisecond parametric protocols (38). It should be noted that depending on the applied pulse duration, the amplitude of the pulses should be adjusted, respectively. The ESOPE protocol with eight square wave pulses of 100 μs typically uses charging voltages of 100–1,000 V to trigger reversible electroporation in the 0.6–1.5 kV/cm electric field range. At the same time, IRE is an irreversible process; thus, the number of pulses and the amplitude of the pulse are increased. Frequently, sequences of more than 80 pulses are used with amplitudes at least two- to threefold higher than the ones used in ECT (39).

In the case of ECT, the ESOPE became a gold standard to be employed in research and clinics, which is still dominant in the context of electrochemotherapy (40–43). However, in the case of IRE, a tendency to reduce the duration of pulses nowadays is observed. Currently, typical unipolar IRE protocols deliver <200-μs pulses to prevent thermal damage and minimize tissue oxidation and muscle contractions (44–46).

In the early 2000s, the time for ultrashort pulsing in the context of electroporation came, and the scientific area of supra-electroporation was formed to develop non-thermal techniques for cell membrane permeabilization. Because of the ultra-short pulse duration, which is shorter than the plasma membrane charging time, the effects of sub-microsecond electric fields differ substantially from conventional micro-millisecond procedures. Due to the dramatic reduction of the pulse duration, the amplitude had to be increased (47). The ultra-short but high-intensity electric field pulses developed voltages across intracellular structures without destructive effects (48–50). It was noted that even short pulses as 3 ns can provoke poration of the lipid bilayer and the externalization of phosphatidylserine (PS), which was demonstrated experimentally and by MD simulations (51). Also, the significant differences in pore size, stability, and distribution between sub-microsecond and microsecond range pulses were indicated (52–55). Up to now, these features are mathematically predicted based on computer simulations, and further studies must be verified by experimental observations. The extremely high amplitude of the nanosecond pulses enabled more uniform permeabilization of the cell membrane, which is advantageous in heterogeneous tissues. Also, with the use of sub-microsecond pulses, modulation of cell death type became possible (i.e., trigger apoptosis), which can be employed to minimize inflammation of the tissue (56, 57). Finally, the potential to use nanosecond pulses in IRE applications for cancer treatment has been revealed (58–62). Nevertheless, the absolute majority of works featuring unipolar nanosecond pulses and electroporation were performed *in vitro*, which can be attributed to the lack of electroporators and technology to generate MV/m electric fields with bigger electrode gap systems required for *in vivo* or clinical applications (25, 63–66). Possibly due to the same reasons, the nanosecond electrochemotherapy was also hardly focused. Nevertheless, with the development of pulsed power technology, the potential of electrochemotherapy by nanosecond pulses has been highlighted in recent years, both *in vitro*, which can be attributed to the lack of electroporators and technology to generate MV/m electric fields with bigger and appropriate electrode gap systems required for *in vivo* or clinical applications. Thus, the electrode problem might also be the reason why nanosecond electrochemotherapy is still beyond the scope of practical application. Lastly, picosecond pulses are also in the field of interest. It is believed that the sub-nanosecond range of pulses (ps) can provoke mainly intracellular effects. Moreover, ultrashort pulse delivery is noninvasive and can be radiated by antennas (67, 68). Experimental studies demonstrated that the picosecond pulse induces the electroporation of intracellular structures while the cell membrane is unaffected (69). Semenov et al. studied picosecond pulses, which are shorter than channel activation time but can activate voltage-gated channels. It was shown that picosecond pulses could cause long-lasting opening of voltage-gated calcium channels by a mechanism different than electroporation (27). In the other study, intense picosecond electric pulses decreased mitochondrial transmembrane potential and induced apoptotic cell death, with caspase-3 and -9 activation (70). In turn, Zamponi et al. highlighted that picosecond electric pulses could be used to break down protein aggregates and affect neural stem cell differentiation, which can be used in neurodegeneration therapies (71). Thus, there is a high potential for ultrashort electric pulses, which can be used for the sensitive activation of the specific channels or organelles in cells.

## Bipolar square wave pulses

In the 2000s, the electroporation techniques using bipolar, also called biphasic pulses, were focused on, while the first works were published in the early 1990s (72). The main motivation for using a more complex waveform (compared to a unipolar sequence) was to decrease the electrolysis and the contamination of the suspension with metal ions, improve the homogeneity of the treatment, and reduce the muscle contractions. It was shown that the bipolar pulses could be successfully used for cell membrane permeabilization, electrochemotherapy, and DNA transfection (73–77). However, considering the increased complexity and limited availability of the bipolar pulse generators until the early 2010s, the technique was hardly popular compared to ESOPE in electrochemotherapy. The benefits of using long (50+ µs) bipolar pulses (compared to ESOPE) were mainly to reduce the muscle electrical excitation, thus better accepted by patients (75, 78–82). Application of bipolar sequences in IRE was more common and used on a routine basis in many clinical applications (28, 83, 84). However, the true potential of bipolar square wave pulses is in the high-frequency domain using short pulses. Switching the pulse polarity with a frequency close to the charging time of plasma membranes can significantly improve the electroporation of heterogeneous tissues (85). Mitigation of impedance changes due to electroporation, and tissue heterogeneity is possible (18, 86, 87). Also, non-thermal ablation without muscle contraction can be ensured (88). With all the positive factors combined, a new electroporation-based technique called High-Frequency Irreversible Electroporation (H-FIRE) was born. However, soon it was determined that reversal of the pulses could weaken the effect (known as bipolar cancellation) due to the membrane depolarization (89, 90). Therefore, accurate tuning of the bipolar sequence is required, and modulation of the treatment *via* the interplay of delay between consequent opposite polarity pulses can be performed (91–93). The phenomenon of bipolar cancellation also formed a base for a new technique called CANCAN (cancellation of bipolar cancellation) when the nanosecond pulses can be structured and synchronized to achieve maximum effect remotely using interference targeting (94). It is particularly useful for targeting deep-seated tumors without collateral damage and is currently in the stage of development.

Nevertheless, the application of high-frequency bipolar pulses is still limited in the context of electrochemotherapy. Firstly, rapid bipolar pulses need larger amplitudes to disrupt cells, similar to longer monopolar pulses (95, 96). Secondly, the electrophoretic component of short bipolar pulses is negligible compared to ESOPE protocols (97). Thus, drug delivery is dominated by passive diffusion. However, when tuned right, the bipolar nanosecond pulse sequences can be as efficient as the standard electroporation protocols (25, 96).

## CANCAN pulses

**Figure 4:**
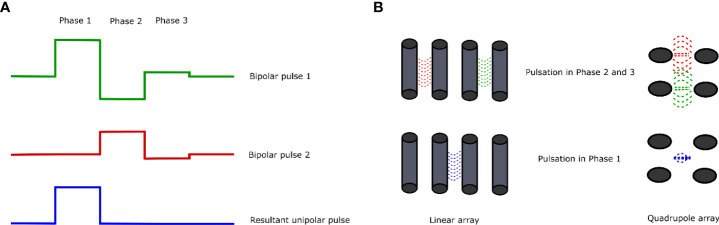
Overview of the CANCAN effect. **(A)** Summation of the bipolar pulses results in the formation of a unipolar pulse with the resultant pulse in Phase 1. **(B)** Two arrangements of electrodes for the study of the CANCAN effect and the effects of pulsation in each of the phases. The left side shows the linear arrangement of the needle electrodes; on the right side, the needle electrodes are arranged in a square—in each case, the distance between the neighboring needles remains constant.

As it was mentioned above, the CANCAN effect stands for the cancellation of bipolar cancellation. The effect is achieved when two bipolar pulses overlap and the array overlay into a unipolar pulse between the system of electrodes (**Figure 4A**) (98). Most studies concerning the CANCAN technique focus on nanoseconds or short microsecond pulses (94). In general, unipolar nanosecond pulses exert several biological effects, i.e., cell death induction on the targeted cells, which is strictly related to membrane permeabilization, and ROS formation as a reaction due to stress (99). However, when the applied pulses remain bipolar, most of the mentioned effects do not occur due to the pulse cancellation from one phase by the pulse from the other phase (89, 90, 100–102). Studies by Sozer and Vernier proved that the mechanisms underlying the cancellation are the multistep process involving the charged species movement, the cellular homoeostatic response effectors, and cell repair mechanisms (103). Pakhomov et al. proved that the effects of bipolar cancellation remain the lowest or even absent at the lowest electric fields, close to the electroporation threshold (94). When the electroporation system requires the use of four electrodes in the specific array, two simultaneous bipolar pulses might be generated that would overlap each other (**Figure 4B**). In this case, the biological effect would arise from the combination of both pulses and not the stand-alone effect of a single bipolar pulsation (98).

Depending on the arrangement of electrodes for the generation of the CANCAN effect, the two most commonly used layouts could be distinguished (**Figure 4B**). The first one includes the quadrupole formation by inserting the electrodes in the rectangle array. In this way, two independent pulses might be generated that overlap in the rectangle’s middle. In this case, the biological effects of PEF treatment would be pronounced in the middle of the system—aside from the electrodes (98). Curiously, studies by Pakhomov et al. showed that even though the permeability of the cells towards Yo-Pro dye should not be highly exposed in the neighboring electrodes, indeed, it is (94). The quadrupole system is suitable for studies concerning targeting regions that do not directly neighbor the electrodes. The second layout of the electrodes is the linear one. This attempt is mainly used for research purposes. It was initially used to study the effects of bipolar pulses combined into a single unipolar pulse outside the electrodes.

Studies by Pakhomov et al. revealed several important pieces of information about the optimization of CANCAN protocol usage (94). The authors compared if the direction of the pulses in the quadrupole layout may modulate the occurrence of the CANCAN effect and found that the pulsation from both systems of electrodes should be delivered in the same direction. Only in this case was the CANCAN effect observed in the middle of the electrodes’ array. The authors also showed that the increase in the pulses’ delivery frequency exacerbated the system’s heating effect. Furthermore, the authors investigated the efficacy of CANCAN induction by the biphasic and triphasic pulses. The experiment showed that the effect is present when biphasic and triphasic pulses are combined without any time gap in their delivery. Namely, Pakhomov et al. delivered a sequence of pulses with a 50-ms gap and observed no CANCAN effect. Conversely, when the unipolar pulse formation arose from the overlap in the simultaneous pulsation with bi- and triphasic pulses, CANCAN was pronounced in the middle of the quadrupole electrodes’ system. The authors further investigated if the rotation of the electrodes would enhance the CANCAN effect and observed the symmetrical pattern of electroporation next to the pair of electrodes that produced the strong triphasic pulses. The amplification of the central CANCAN peak was observed as well. The authors also analyzed the effects of first phase pulse intensity and the effects of the pulse number on the uptake of YO-PRO-1 in the rotatory electrodes’ system. The study revealed that the increase in both parameters induces higher dye uptake in the center of the electrodes’ layout. Finally, the authors showed that the increase in the number of CANCAN stimuli induced an even cell killing area in the region encompassed by the electrodes and not only partially in the middle of the electrodes’ array. Gianulis et al. present several challenges in the application of CANCAN pulses (98). Namely, electric pulses can be distorted when traveling in various human fluid spaces, so the theoretical and realistic courses of electric pulses vary in time and space. Each medium in which we pass the electric current has a different conductivity (104); therefore, it is extremely hard to synchronize the pulses in time and space. Moreover, the electric field is higher near the electrodes; thus, tissue damage near the electrodes might occur at a lower applied voltage. The problem arises when we want to affect deep tissues while preventing tissues nearby electrodes from being damaged. Also, the effective distance from the electrodes for cell stimulation has to be enhanced. Recent advances came from Roth et al., who used deep H-coils, doubling the distance for deep tissue stimulation (105). The attempt, however, involves less precise targeting of the tissues. The other type of electrode setup involves the cathode ring surrounding an anode disc electrode. This arrangement may be used to stimulate brain tissue (106).

## Asymmetric pulses

**Figure 5:**
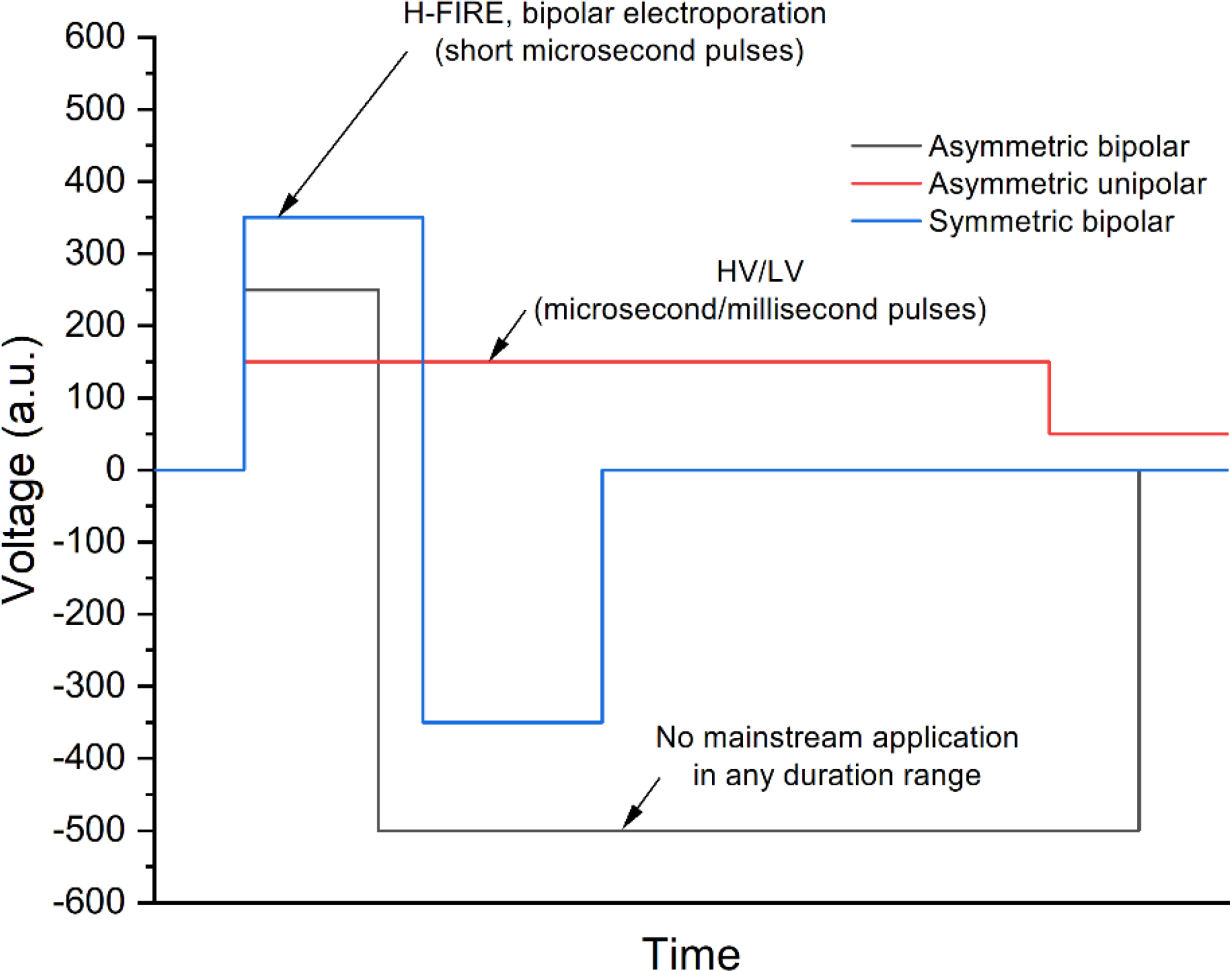
The examples of asymmetric and symmetric waveforms.

An asymmetric pulse means that the device can generate pulses of a positive and/or negative polarity where the pulse duration and pulse amplitude of a positive and/or negative polarity can be set independently, and the pause between both polarities can be changed (see **Figure 5**). The asymmetry can be ensured both in the intensity and in the duration space within the operation range of the device.

One of the most established techniques that employ asymmetric pulses is the high-voltage low-voltage methodology (HV/LV) for gene delivery (107–109). This technique uses the high-voltage microsecond range pulse (higher than the electroporation threshold) for permeabilization of cells and the low voltage (millisecond range pulse) for electrophoretic force induction. In such a way, the efficacy of transfection can be significantly improved since the delivery of DNA is dependent on the electrophoretic component of the treatment (110). The HV/LV method is hardly used in any other electroporation-based context. At the same time, the asymmetric bipolar pulses did not find any mainstream application yet. In the microsecond range, there are efforts to use such pulses for electrical isolation of cardiac tissue in atrial arrhythmias. Asymmetric high-frequency waveforms generate abrasions in cardiac tissue. Asymmetric high-frequency waveforms can create deeper lesions than a symmetric waveform with the same energy or a symmetric waveform with the same charge (111). Sano et al. demonstrated that the lethal threshold for H-FIRE treatments is also affected by the symmetry of the pulses. Asymmetric waveforms have significantly lower lethal thresholds than equivalent energy symmetric waveforms. Asymmetric high-frequency irreversible electroporation (H-FIRE) can be used to generate ablation volumes corresponding to standard IRE protocols (112). We speculate that the lack of works focusing on the asymmetric bipolar pulses is partly influenced by the lack of commercial and non-commercial generators able to generate such pulses. Nevertheless, the efforts to develop such technology are performed in a systemic manner. For example, Grainys et al. have developed a high-voltage, bipolar electroporator that generates single or multiple, symmetrical or asymmetrical, high-power square wave microsecond pulses up to ±1 kV and 100 A (113). Levkov et al. reported on a PEF system consisting of a high-voltage generator with an asymmetric voltage multiplying architecture and a treatment chamber with sliding electrodes for marine macroalgae electroporation (114). The system enables pulses of up to 4 kV and 1 kA with a pulse duration between 1 μs and 100 μs.

At the same time, the nanosecond asymmetric pulse range experimentally is hardly covered at all due to even more extreme technological challenges. However, in the past several years, at least three new systems have been reported. In 2021, Pirc et al. presented a prototype of the asymmetric bipolar pulse generator that can generate 4-kV pulses with 131-A maximal current, and duration as low as 200 ns (25). Kandratsyeu et al. presented a 6.5-kV asymmetric pulse generating device with a minimum pulse duration of 100 ns (115). Novickij et al. developed a device for 65 ns–100 µs asymmetric pulse generation in 2022 (26). It could be speculated that the high-energy asymmetric nanosecond pulses will elicit stronger and more selective effects on intracellular membranes than symmetrical pulses.

## The effects of pulse repetition rate

The importance of pulse amplitude and duration in the context of electroporation cannot be overestimated. However, the frequency of the delivered pulses forms an additional degree of freedom in parametric protocol design. In the microsecond range, frequency manipulation is rarely performed, with most protocols relying on low frequency (1–10 Hz). The main reason is the lack of electrotransfer effectiveness increase in the kilohertz range and the problems associated with excitation of the nerve fibers, which causes higher torque of muscle contraction (78, 116). Operating with extremely slow pulse delivery protocols (0.1–1 Hz) triggers a phenomenon known as “electrosensitization” when the long delays between the pulse bursts influence a higher uptake of molecules (117, 118). The improved effectiveness of the treatment was confirmed *in vivo*, too (119). However, the technique is not commonly used yet. With the increase of frequency from 1 to 10 Hz, the density of the pores on the cellular membrane increases (120), resulting in the higher uptake of fluorescent markers for nanosecond and microsecond pulses (78, 121). Romeo et al. have also shown that nanosecond pulses delivered at 1-kHz repetition frequency result in higher YO-PRO-1 uptake compared to the 1-Hz protocol (122), while Vernier et al. have confirmed that the increase of repetition frequency to 10 kHz further improves the molecular electrotransfer by nanosecond electric field pulses (123). Therefore, an increase of repetition frequency in the 1 Hz–10 kHz range is beneficial for nanosecond PEF-based procedures to ensure a faster and more efficient (electrotransfer-wise) treatment without significant changes in the intensity of muscle contractions. However, there are hardly any benefits for longer microsecond (i.e., 100 µs)-based procedures since the 1-Hz and kilohertz protocols can be used interchangeably with minor changes in the treatment outcome (124). The microsecond pulses are also hardly used in the middle kilohertz range (10–100 kHz), while the effectiveness of nanosecond pulse bursts is reduced (121, 125, 126). The exceptionally high-frequency (100 kHz–10 MHz) pulse repetition range is the least covered in scientific literature due to a lack of technological infrastructure. Nevertheless, a further increase in repetition frequency of nanosecond pulses triggers a new high-frequency (500 kHz–1 MHz) phenomenon of transmembrane potential accumulation resulting in a significantly increased electroporation efficiency (121, 125, 127). It was shown that it is possible to achieve a threshold repetition frequency when the discharging [transmembrane potential (TMP) relaxation] time of the membrane is higher than the delay between the pulses; thus, the TMP starts to accumulate throughout the burst (121, 128). The phenomenon in some studies is referred to as “MHz pulse compression” (129) and allows one to significantly lower the electroporation threshold using the pulse repetition frequency modulation. Based on *in silico* observations, the high repetitions rate pulses can modulate the size of the pores after electroporation, which is important both in drug delivery and in irreversible electroporation (130, 131). The 10-MHz+ range pulses in the context of the electroporation are not covered experimentally yet; however, the first works presenting theoretical guidance start to appear (132). It is predicted that the pore formation time becomes shorter when the symmetrical bipolar picosecond pulse train repetition frequency decreases (from the terahertz to megahertz range). The interfacial water (i.e., water dipole moment) is believed to play a key role in the high-frequency electroporation process (132). The same tendency is theoretically predicted for square wave unipolar bursts in the sub-terahertz repetition frequency region (133).

To summarize, the pulse repetition frequency is an important parameter for shorter pulses; however, for longer (i.e., 100 µs) pulses—the influence is minor and typically 1- or 10-Hz protocols are used.

## Conclusions

Summarizing, we can conclude that pulse shape is crucial for electroporation, and accurate metrology is required for treatment planning and results interpretation. Depending on the pulse intensity and duration, the effects of other parameters such as pulse number or repetition frequency will be altered. The load of the generator should also be considered, to prevent a significant transient process, which, if not compensated, will significantly alter the cellular response. Considering all the factors and the different susceptibility of cells to electroporation, an individual approach should be performed depending on the application. Finally, it is concluded that there is a tendency to move toward the shorter pulse duration range, which is a logical evolution of microsecond range electroporation. However, shorter pulses are harder to generate, control, and measure, requiring higher interdisciplinary skills; thus, the electroporation community needs to adapt too.

## Author contributions

JK and VN deigned the concept of the review, prepared and validated manuscript, NR and WS prepared Cancan and asymmetric pulses related description. All authors contributed to the article and approved the submitted version.

## Funding

Funding was provided by the Polish National Centre of Science of DAINA 2 (2020/38/L/NZ7/00342; PI: JK) and the Research Council of Lithuania grant (Nr. S-LL-21-4, PI: VN).

## Conflict of interest

The authors declare that the research was conducted in the absence of any commercial or financial relationships that could be construed as a potential conflict of interest.

## Publisher’s note

All claims expressed in this article are solely those of the authors and do not necessarily represent those of their affiliated organizations, or those of the publisher, the editors and the reviewers. Any product that may be evaluated in this article, or claim that may be made by its manufacturer, is not guaranteed or endorsed by the publisher.
